# Neurogenetic plasticity and sex influence the link between corticolimbic structural connectivity and trait anxiety

**DOI:** 10.1038/s41598-017-11497-2

**Published:** 2017-09-08

**Authors:** M. Justin Kim, Reut Avinun, Annchen R. Knodt, Spenser R. Radtke, Ahmad R. Hariri

**Affiliations:** Laboratory of NeuroGenetics, Department of Psychology and Neuroscience, Duke University, Durham, NC 27708 USA

## Abstract

Corticolimbic pathways connecting the amygdala and ventral prefrontal cortex (vPFC) are linked with trait anxiety, but it remains unclear what potential genetic moderators contribute to this association. We sought to address this by examining the inter-individual variability in neuroplasticity as modeled by a functional polymorphism (rs6265) in the human gene for brain derived neurotrophic factor (*BDNF*). Amygdala-vPFC pathway fractional anisotropy (FA) from 669 diffusion magnetic resonance images was used to examine associations with trait anxiety as a function of rs6265 genotype. We first replicated the inverse correlation between trait anxiety and amygdala-vPFC pathway FA in women. Furthermore, we found a moderating influence of rs6265 genotype such that the association between trait anxiety and right amygdala-vPFC pathway FA was strongest in women carrying the Met allele, which is linked with decreased activity-dependent neuroplasticity. Results indicate that the microstructural integrity of pathways supporting communication between the amygdala and vPFC help shape the expression of trait anxiety in women, and that this association is further modulated by genetically driven variability in neuroplasticity.

## Introduction

Neurobiological theories of anxiety postulate that an interconnected network of brain regions, which typically includes the amygdala^1^, the medial prefrontal cortex^2^, and the bed nucleus of the stria terminalis^3^, are central to the generation and regulation of anxious states. Of particular importance for the experience of anxiety are the dynamic reciprocal interactions between the amygdala and medial prefrontal cortex^4, 5^. These brain regions are strongly implicated not only in the normative experience of anxiety but also in its pathological expression as anxiety disorders, which are often associated with structural and functional differences in these regions^6–10^.

One method of assessing the potential association between the aforementioned neural circuitry and trait anxiety is to determine the degree to which the structural integrity of pathways between the amygdala and medial prefrontal cortex is related to individual differences in trait anxiety. A number of studies have utilized diffusion magnetic resonance imaging (dMRI) – a noninvasive neuroimaging method that measures water diffusivity in brain tissue *in vivo* – to show that the structural connectivity of the pathways connecting the amygdala and the ventral prefrontal cortex (vPFC) is inversely associated with trait anxiety^11–14^. Complementing these structural findings, studies using functional magnetic resonance imaging (fMRI) have demonstrated that the functional connectivity between the amygdala and the vPFC is inversely correlated with self-reported levels of anxiety^15–18^. This convergent evidence suggests that increased capacity for efficient communication between the amygdala and vPFC – represented by both greater functional and stronger structural connectivity – is associated with lesser trait anxiety^9^.

While the inverse association between amygdala-vPFC structural connectivity and anxiety is further supported by studies of clinical populations such as generalized anxiety disorder^19, 20^ and social anxiety disorder^21, 22^, comparison of available studies examining trait anxiety in non-clinical samples has been inconsistent, possibly related to inadequate power to detect small effects as well as to explore the influence of potential moderators such as sex and age^23–25^. Our recent study^13^ aimed to address this issue by examining these links in a relatively large sample (*n* = 275) comprised of three independent datasets. In addition to observing a significant inverse association between amygdala-vPFC structural connectivity and trait anxiety, our work further revealed sex differences wherein the negative correlation was primarily driven by women. While some prior studies analyzing data across men and women did report an overall significant inverse association^12, 14^, our prior work offered a possibility that sex differences, along with potential age differences, may be partially responsible for the mixed findings in the literature.

Here, we use a large dataset (*n* = 669) from the Duke Neurogenetics Study (DNS) to further examine the moderating role of sex in the expression of trait anxiety as a function of amygdala-vPFC structural connectivity. Given the availability of genetic data in the DNS, we further explored the moderating effect of putative inter-individual variability in neuroplasticity on any observed associations. Specifically, we leverage a functional polymorphism (rs6265) in the human brain derived neurotrophic factor gene (*BDNF*) to model individual differences in activity dependent plasticity^26^ as well as neurodevelopment^27^, which both theoretically impact the structure and function of our target amygdala-vPFC pathway. Moreover, *BDNF* rs6265 has been linked with anxiety-like phenotypes including fear extinction as well as clinical disorder^28–30^. Based on the literature reviewed above, we hypothesized that the association between amygdala-vPFC structural connectivity and trait anxiety would be stronger in women compared to men as well as in carriers of the BDNF rs6265 Met allele, which is associated with relatively impaired activity-dependent plasticity and fear extinction as well as risk for anxiety disorders.

## Results

### Demographic and behavioral characteristics

The 669 participants self-reported as being Caucasian (*n* = 338, 50.5%), Black or African American (*n* = 76, 11.4%), Asian (*n* = 180, 26.9%), American Indian or Alaska Native (*n* = 2, 0.3%), Multiracial (*n* = 52, 7.8%), and Other (*n* = 21, 3.1%), as well as Hispanic/Latino (*n* = 125, 18.7%). Average trait anxiety scores were 37.7 (±9.3), and there were no significant effects of sex, genotype, or their interaction. Out of the 669 participants, 128 were diagnosed with past or current DSM-IV diagnosis (Table 1). The remaining 541 participants were free of past or current diagnosis.

**Table 1:** Diagnoses for past or current mental disorders in the total study sample (*n* = 128).

Diagnosis	Number of Participants
Major depressive disorder	26
Bipolar disorder I or II	5
Bipolar disorder – Not otherwise specified	11
Hypomanic episode	12
Panic disorder	8
Agoraphobia	10
Social anxiety disorder	7
Generalized anxiety disorder	13
Obsessive-compulsive disorder	6
Posttraumatic stress disorder	1
Alcohol abuse/dependence	75
Marijuana abuse/dependence	23
Eating disorder	5
Antisocial personality disorder	1
Borderline personality disorder	1

The sum of the individual diagnoses is higher than the number of participants with mental disorders because a subsample of them has multiple comorbid diagnoses.

The subgroup of 541 healthy participants self-reported as being Caucasian (*n* = 261, 48.2%), Black or African American (*n* = 64, 11.8%), Asian (*n* = 153, 28.3%), American Indian or Alaska Native (*n* = 2, 0.4%), Multiracial (*n* = 43, 8%), and Other (*n* = 18, 3.3%), as well as Hispanic/Latino (*n* = 105, 19.4%). Average trait anxiety scores were 36.8 (±8.6), and there were no significant effects of sex, genotype, or their interaction.

### Amygdala-vPFC structural connectivity and trait anxiety

Hierarchical regression analyses of data from all participants (*n* = 669) revealed that adding amygdala-vPFC pathway fractional anisotropy (FA) in the second step did not significantly improve the model from the first step that included age, sex, and head motion, in predicting self-reported trait anxiety (first step: *R* ^2^ = 0.007, *F*(3,665) = 1.489, *p* = 0.216, second step, right pathway: Δ*R* ^2^ = 0.001, Δ*F*(1,664) = 0.482, *β* = −0.029, *p* = 0.488; second step, left pathway: Δ*R* ^2^ = 0.003, Δ*F*(1,665) = 1.924, *β* = −0.057, *p* = 0.166). Analyses restricted to healthy participants (*n* = 541) revealed weak yet significant inverse associations (first step: *R* ^2^ = 0.01, *F*(3,537) = 1.762, *p* = 0.153, second step, right pathway: Δ*R* ^2^ = 0.008, Δ*F*(1,536) = 4.213, *β* = −0.093, *p* = 0.041; second step, left pathway: Δ*R* ^2^ = 0.011, Δ*F*(1,536) = 5.887, *β* = −0.11, *p* = 0.016). All participants included in the current study had an acceptable degree of head motion (average range: 0.18–0.64 mm). In both analyses, average FA of the cingulum bundle and the sagittal stratum, which were selected as control pathways, were not significantly associated with trait anxiety.

### Sex difference in the present brain-anxiety association

**Figure 1:**
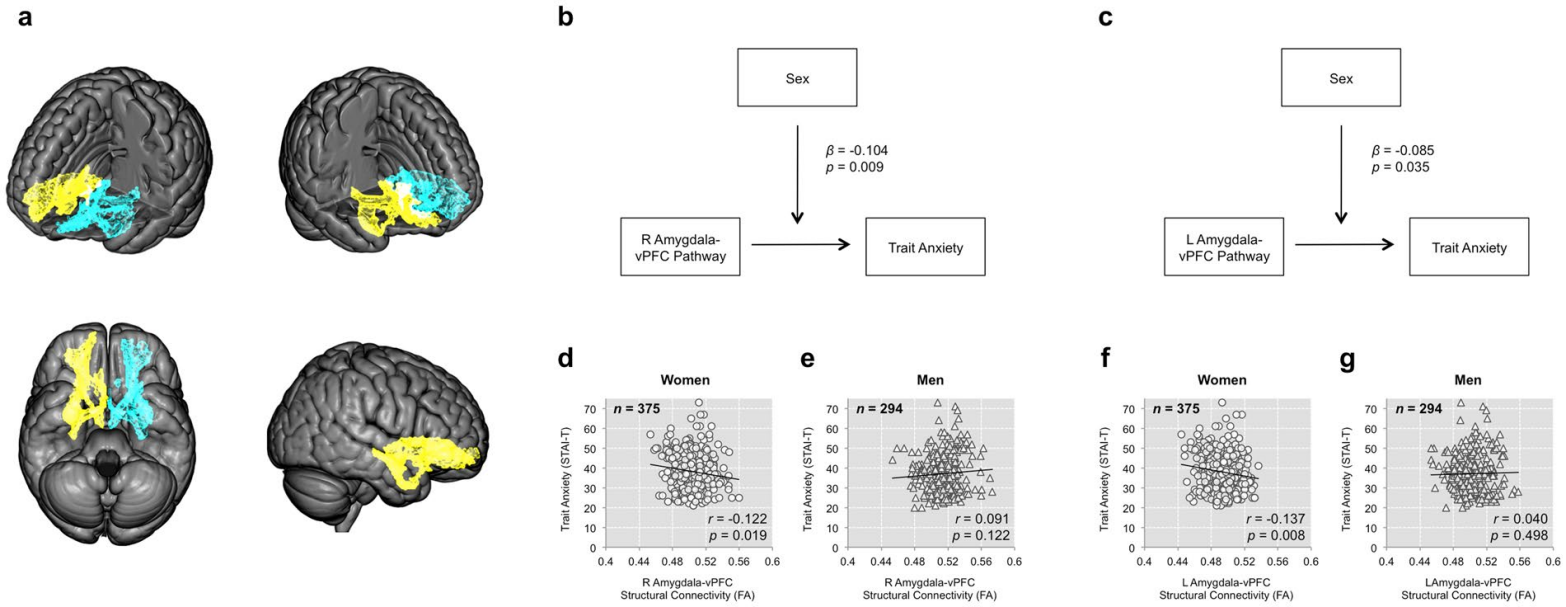
Moderating effect of sex. (**a**) An illustration of the right (yellow) and left (cyan) amygdala-vPFC pathway masks, defined using probabilistic tractography data from an independent group of samples (*n* = 71)^13^. (**b**) The association between the right amygdala-vPFC pathway strength and trait anxiety was significantly moderated by sex. (**c)** The same moderating effect of sex was observed for the left amygdala-vPFC pathway. Both *p*-values were below the FDR-corrected threshold of *q* < 0.05. (**d**,**e**) Significant inverse correlations between the right amygdala-vPFC pathway strength and trait anxiety were observed in women but not men. (**f**,**g**) The same effect was found in the left amygdala-vPFC pathway. All *p*-values were below the FDR-corrected threshold of *q* < 0.05. Correlation coefficients were calculated controlling for the effects of age and head motion.

A significant moderating effect of sex was observed for the amygdala-vPFC pathways in predicting trait anxiety, after controlling for the effects of age and head motion (right: Δ*R* ^2^ = 0.01, *F*(1,663) = 6.911, *β* = −0.104, *p* = 0.009; left: Δ*R* ^2^ = 0.007, *F*(1,663) = 4.476, *β* = −0.085, *p* = 0.035; Fig. 1b,c). Upon closer inspection, we found that the sex difference was driven by significant inverse associations in women (first step: *R* ^2^ = 0.008, *F*(2,372) = 1.486, *p* = 0.228, second step, right pathway: Δ*R* ^2^ = 0.015, Δ*F*(1,371) = 5.564, *β* = −0.127, *p* = 0.019; second step, left pathway: Δ*R* ^2^ = 0.019, Δ*F*(1,371) = 7.063, *β* = −0.141, *p* = 0.008; *q*s < 0.05), but not in men (Fig. 1d–g).

Results were consistent when the analysis only included healthy participants, as a significant moderating effect of sex was observed for the amygdala-vPFC pathways in predicting trait anxiety (right: Δ*R* ^2^ = 0.01, *F*(1,535) = 5.273, *β* = −0.094, *p* = 0.022; left: Δ*R* ^2^ = 0.009, *F*(1,535) = 4.902, *β* = −0.091, *p* = 0.027). Similarly, this sex difference was driven by significant inverse association in women (first step: *R* ^2^ = 0.008, *F*(2,315) = 1.296, *p* = 0.275, second step, right pathway: Δ*R* ^2^ = 0.027, Δ*F*(1,314) = 8.937, *β* = −0.176, *p* = 0.003; second step, left pathway: Δ*R* ^2^ = 0.034, Δ*F*(1,314) = 11.011, *β* = −0.19, *p* = 0.001; *q*s < 0.05), but not in men. For all moderator analyses of sex, there were no significant results associated with the cingulum bundle or the sagittal stratum.

### Moderating effect of rs6265 genotype on sex-dependent brain-anxiety association

There were no overall significant effects of rs6265 genotype on brain-anxiety associations. However, a significant moderating effect of rs6265 genotype × sex interaction was observed for the right amygdala-vPFC pathway in predicting trait anxiety. This association was robust to controlling for the effects of age, ancestry, and head motion (Δ*R* ^2^ = 0.016, *F*(3,655) = 3.709, *p* = 0.012). Unpacking the interaction, *post hoc* hierarchical regression tests showed that this effect was driven by a significant inverse association in women carrying the Met allele (first step: *R* ^2^ = 0.128, *F*(6,131) = 3.203, *p* = 0.006, second step: Δ*R* ^2^ = 0.046, Δ*F*(1,130) = 7.21, *β* = −0.227, *p* = 0.008) and a positive association in men carrying the Met allele (first step: *R* ^2^ = 0.112, *F*(6,113) = 2.387, *p* = 0.033, second step: Δ*R* ^2^ = 0.04, Δ*F*(1,112) = 5.272, *β* = 0.214, *p* = 0.024; *q*s < 0.05), but not for all other groups. This moderating effect was not observed in the left amygdala-vPFC pathway.

**Figure 2:**
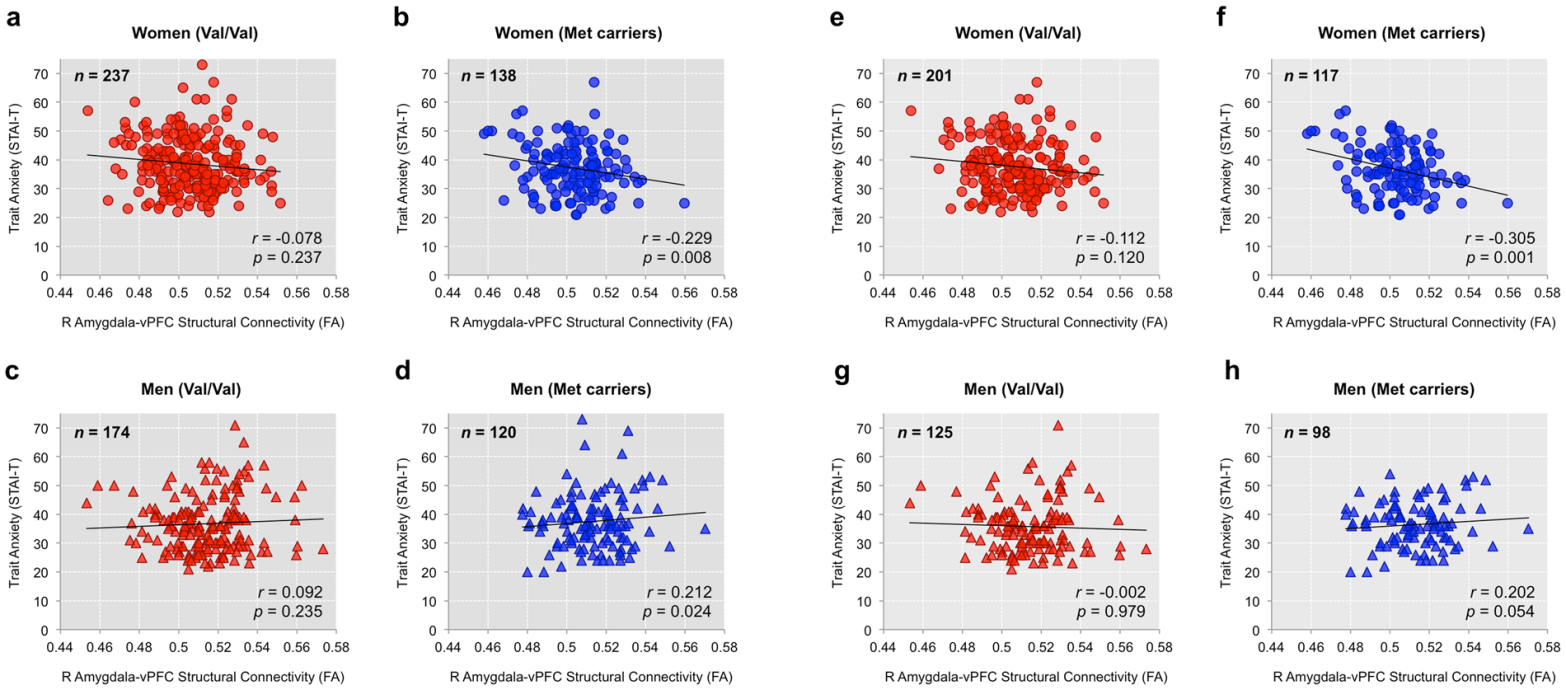
Moderating effect of rs6265 genotype on sex-dependent brain-anxiety association. (**a**–**d**) Scatterplots depicting a moderating effect sex × BDNF genotype interaction for all participants (*n* = 669). Of the four possible sex × BDNF genotype pairs, only the women Met-allele carriers (blue circle) displayed a significant inverse correlation between the microstructural integrity of the right amygdala-vPFC pathway and trait anxiety. Conversely, men Met-allele carriers (blue triangle) showed a positive correlation. Both *p*-values were below the FDR-corrected threshold of *q* < 0.05. (**e**–**h**) When the analysis was limited to healthy participants only (*n* = 541), only the women Met-allele carriers (blue circle) displayed a significant inverse correlation between the microstructural integrity of the right amygdala-vPFC pathway and trait anxiety. The *p*-value was below the FDR-corrected threshold of *q* < 0.05. All correlation coefficients were calculated controlling for the effects of age, ancestry, and head motion.

This interaction effect between rs6265 genotype and sex on the right amygdala-vPFC pathway remained significant when the analysis was restricted to healthy participants (ΔR^2^ = 0.02, F(3,527) = 3.886, p = 0.009). *Post hoc* tests showed that a significant inverse association was only found in women with the Met allele (first step: *R* ^2^ = 0.182, *F*(6,110) = 4.086, *p* = 0.001, second step: Δ*R* ^2^ = 0.076, Δ*F*(1,109) = 11.18, *β* = −0.29, *p* = 0.001, *q* < 0.05), but not for all other groups (Fig. 2). The positive association in men carrying the Met allele was no longer statistically significant (first step: *R* ^2^ = 0.087, *F*(6,91) = 1.438, *p* = 0.209, second step: Δ*R* ^2^ = 0.037, Δ*F*(1,90) = 3.826, *β* = 0.208*, p* = 0.054). For all analyses, average FA of the cingulum or the sagittal striatum were not significantly associated with trait anxiety in any of the BDNF genotype × sex subgroups. For completeness, a summary of the pairwise Pearson correlation tests for all sub groups is provided in Supplementary Tables S3 and S4.

## Discussion

Using a large multimodal dataset from the Duke Neurogenetics Study, we demonstrate that the expression of trait anxiety as a function of the microstructural integrity of a prominent pathway linking the amygdala and vPFC is moderated by sex and a functional genetic polymorphism associated with neuroplasticity. Replicating our prior work^13^, we found an inverse correlation between amygdala-vPFC FA values and self-reported trait anxiety in women but not men. Furthermore, we demonstrate that this association in women is specific to Met allele carriers of *BDNF* rs6265, which is associated with impaired activity-dependent plasticity as well as multiple anxiety-related phenotypes including impoverished fear extinction. The latter is particularly interesting as fear extinction is largely mediated by dynamic top-down regulation of the amygdala by the medial PFC through the structural pathway targeted in our current study^7–10^. Overall, our findings support the general proposal that individuals with an increased capacity for crosstalk between the amygdala and the vPFC may respond more adaptively to stressful experiences in the explicit form of lower trait anxiety^9^.

While the exact nature of the sex-specific association between amygdala-vPFC and trait anxiety remains to be determined, it is likely that both psychosocial (e.g., some young men may be more reluctant than women to report their own levels of trait anxiety^31^) and biological (e.g., prenatal stress may lead to increased anxiety-like behaviors in female versus male rats^32^) mechanisms contribute to the observed differences between men and women. We do note that these particular sex differences in dMRI studies of brain-anxiety associations have emerged only recently^13^, and that not all previous studies reporting an inverse correlation between trait anxiety and amygdala-vPFC structural connectivity observed moderating effects of sex^12, 14^. Regardless, the current findings from our large sample, which had better power to detect small effects than any prior work, along with previously reported sex-specific effects buttress the link between trait anxiety and the structural connectivity of the amygdala-vPFC pathways in women but not men. These structural findings are further corroborated by parallel findings from fMRI research, where a similar inverse correlation between amygdala-vPFC resting state functional connectivity and anxiety was found to be specific to women^16^. These converging data on the neurobiological correlates of trait anxiety could usefully inform ongoing efforts to better treat and, ultimately, prevent pathological anxiety as a function of sex-specific pathways.

A novel contribution of our study to the extant literature is the demonstration that a common functional polymorphism associated with activity dependent neuroplasticity further moderates the sex-specific association between amygdala-vPFC structural connectivity and trait anxiety. We found that women who were carriers of the BDNF rs6265 Met allele exhibited a negative association between the right amygdala-vPFC pathway and anxiety. Conversely, Val/Val homozygotes of either sex did not show significant correlations. An underlying molecular mechanism for these sex-specific genotype differences may involve transcriptional regulation of BDNF through estrogen response elements^33^. Importantly, these effects were consistently observed across all our analyses (i.e., all participants, only participants with no history of clinical diagnosis, and only non-Hispanic Caucasians). However, as the positive correlation observed in men who were Met allele carries was not predicted, we refrain from further speculation and await replication in future research. Another inquiry that could be addressed in future investigations is the potential issue of laterality, since in the present data the moderating effect of rs6265 genotype × sex interaction was found only for the right amygdala-vPFC pathway in predicting trait anxiety. We also note the relatively small effect sizes reported here are consistent with previous imaging genetics research^34–36^, potentially representing the complex and polygenic nature of variability that manifests in the brain and behavior. Finally, while our data showed relative specificity of the observed effects within the amygdala-vPFC pathway, future studies utilizing dMRI with higher angular sampling resolution could further examine white matter pathways that connect the amygdala with more dorsal regions of the prefrontal cortex^12, 37^ in the context of *BDNF*, sex differences, and trait anxiety. Overall, taking into consideration fMRI findings showing that Met allele carriers are characterized by an impaired ability to extinguish a conditioned fear response and by atypical functional activity of the amygdala and the vPFC^29^, our dMRI findings further highlight the importance of the Met allele in the context of the amygdala-vPFC circuitry and anxiety.

Another factor to consider is the neurodevelopmental trajectory of the amygdala-vPFC circuitry. The uncinate fasciculus is among the slowest to mature, as it has been suggested that this corticolimbic white matter fiber does not reach maturity until the third decade of life^38^. These structural findings are complemented by functional studies, in which the pattern of amygdala-vPFC connectivity has been shown to change similarly across development^39, 40^. A recent study reported a positive correlation between the structural connectivity of the amygdala-vPFC pathway and anxiety/depression symptoms in late childhood (~11 years of age), demonstrating the importance of age in assessing the present brain-anxiety association^41^. The potential significance of age is further highlighted given the suggested role of *BDNF* rs6265 in the expression of anxiety as a function of gene by environment interaction across development^42^. Considering our findings were derived from young adults (18–22 years of age), understanding how the present association shifts across development, both at a younger and older age, represents an important next step.

## Methods

### Participants

A total of 669 undergraduate students (375 women, age range 18–22 years, mean age = 19.6 years) who successfully completed the DNS between January 29^th^, 2010 and November 12^th^, 2013 had available dMRI (two scans), behavioral, and genetic data for our analyses. The DNS aims to assess the associations among a wide range of behavioral, neural, and genetic variables in a large sample of young adults, with one of the core goals being to establish a link between these various phenotypes and psychopathology. For this reason, participants were not initially excluded based on past or current diagnosis of Axis I or select Axis II disorders (detailed diagnosis information is summarized in Supplementary Table S1) according to the *Diagnostic and Statistical Manual of Mental Disorders, Fourth Edition* ^43^. However, given that previous studies assessing the relationship between structural connectivity of the amygdala-vPFC pathway and trait anxiety did exclude for past or current diagnosis of mental disorders^11–14^, analyses on a subset of 541 participants (318 women, age range 18–22 years, mean age = 19.6 years) free of such diagnoses are also reported. In addition, all of the analyses were further conducted in non-Hispanic Caucasian participants (total *n* = 276; healthy *n* = 212), the largest homogenous racial/ethnic subgroup available, to minimize potential confounding effects of genetic background on any observed associations (Supplementary Results).

This study was approved by the Duke University Medical Center Institutional Review Board, and all experiments were performed in accordance to its guidelines. Prior to the study, all participants provided informed consent. To be eligible for DNS, all participants were free of the following conditions: 1) medical diagnoses of cancer, stroke, head injury with loss of consciousness, untreated migraine headaches, diabetes requiring insulin treatment, chronic kidney, or liver disease; 2) use of psychotropic, glucocorticoid, or hypolipidemic medication; and 3) conditions affecting cerebral blood flow and metabolism (e.g., hypertension). Each participant’s self-reported levels of trait anxiety were assessed using the State-Trait Anxiety Inventory-Trait Version (STAI-T)^44^. We note that while STAI-T is known to actually index both anxiety and depression^45^ and thus the scores may better reflect negative affect broadly^7^, the term “trait anxiety” is used here to facilitate comparison with previous studies using this measure.

### Image acquisition

Diffusion-weighted and high-resolution anatomical T1-weighted magnetic resonance imaging scans were acquired using an 8-channel head coil for parallel imaging on one of two identical research-dedicated GE MR750 3 T scanners (GE Healthcare) at the Duke-UNC Brain Imaging and Analysis Center. Following an ASSET calibration scan, two 2-min 50-s diffusion-weighted images were acquired, providing full brain coverage with 2 mm isotropic resolution and 15 diffusion-weighted directions (echo time (TE) = 84.9 ms, repetition time (TR) = 10,000 ms, *b* value = 1,000 s/mm^2^, field of view (FOV) = 240 mm, flip angle = 90°, matrix = 128 × 128, slice thickness = 2 mm). High-resolution anatomical T1-weighted MRI data were obtained using a 3D Ax FSPGR BRAVO sequence (TE = 3.22 ms, TR = 8.148 ms, FOV = 240 mm, flip angle = 12°, 162 sagittal slices, matrix = 256 × 256, slice thickness = 1 mm with no gap).

### Diffusion MRI analysis

All dMRI data were preprocessed in accordance with the protocol developed by the Enhancing Neuro Imaging Genetics through Meta-Analysis consortium (ENIGMA; http://enigma.ini.usc.edu/protocols/dti-protocols/). Raw diffusion-weighted images were corrected for eddy current, and aligned to the non-diffusion-weighted (b0) image using linear registration in order to correct for head motion. Volume-by-volume head motion was quantified by calculating the root mean square (RMS) deviation of the six motion parameters (three translation and three rotation components), for each pair of consecutive diffusion-weighted brain volumes. The resulting volume-by-volume RMS deviation values were averaged across all images, yielding a summary statistic of head motion for each participant, which were used as a covariate of no interest in all subsequent group level analyses. Next, following skull stripping, diffusion tensor models were fit at each voxel using the FSL software package and its Diffusion Toolbox^46, 47^, generating a whole-brain fractional anisotropy (FA) image for each participant. For each participant, an average of the two FA images were produced and these images were then subjected to a tract-based spatial statistics (TBSS) procedure, implemented in FSL^48^. TBSS analysis entails the realignment of each individual FA images to a standard FA template in Montreal Neurological Institute (MNI) space using nonlinear registration (FNIRT). Next, a group-mean FA image was generated and subsequently thinned at a threshold of FA >0.2 to extract the mean FA skeleton, which is assumed to represent the center of the white matter fiber tracts common to the group. Then, individual FA images were projected onto the mean FA skeleton, searching for maximal FA values perpendicular to the skeleton. The resulting individual FA skeletons were used to extract average FA values for our *a priori* regions of interest.

### Region-of-interest analysis

Given that our goal was to first replicate the moderating effect of sex on the inverse correlation between trait anxiety and the microstructural integrity of the amygdala-vPFC pathways, we adopted a region-of-interest (ROI) approach. The bilateral amygdala-vPFC pathways from our previous dMRI study^13^ were used as *a priori* ROI masks for the current study. These amygdala-vPFC pathways were defined using probabilistic tractography on 71 dMRI scans acquired with a higher angular sampling resolution (61 noncollinear diffusion directions), and stored in standard MNI space. This pathway includes parts of the uncinate fasciculus, a major white matter fiber tract that connects the inferior frontal cortex and the anterior temporal lobe^49^. In the previous study^13^, the microstructural integrity of these pathways – indexed by computing the average FA values – was inversely correlated with self-reported levels of trait anxiety in multiple independent datasets. Furthermore, the right amygdala-vPFC pathways showed a significant moderating effect of sex. We note that in the previous study^13^, the amygdala-vPFC pathways were further delineated into a medial and a lateral subdivision; but since the outcomes of that study showed no evidence to support medial vs. lateral differences with respect to trait anxiety, the two subdivisions were combined to create the ROI mask for the current study (Fig. 1a). These ROIs were used to mask each participant’s FA skeleton maps, and then the average FA values for the left and right amygdala-vPFC pathways were extracted on a subject-by-subject basis for further statistical analyses.

This ROI approach has a number of advantages. First, using ROI masks derived from an independent dataset allows for rigorous statistical tests that help reduce Type I error. Second, the amygdala-vPFC pathway masks were derived from dMRI scans with a substantially higher number of diffusion gradients than the current dataset (61 vs. 15), with higher angular sampling resolution enabling a more sensitive detection of white matter tracts through tractography. Third, this approach is able to further validate the generalizability and usefulness of these amygdala-vPFC pathway masks, which could serve as a quick and reliable method for future studies that aim to establish a link between dMRI measures and behavior.

Finally, to rule out the possibility that corticolimbic white matter fiber tracts other than the amygdala-vPFC pathways may be correlated with trait anxiety, the cingulum bundle was chosen to serve as a control pathway. The bilateral cingulum bundle was defined using the Johns Hopkins University DTI-based white matter atlas^50^, and its average FA was computed following the same procedures described above. Similarly, the sagittal stratum mask (which includes the inferior longitudinal fasciculus and the inferior fronto-occipital fasciculus) was chosen as a control pathway from the Johns Hopkins University DTI-based white matter atlas^50^, which approximates the anatomical connections between the amygdala and the visual cortical areas documented in a previous dMRI study of anxiety^12^.

### DNA extraction and genotyping

Genotyping was conducted by 23andMe, Inc. (Mountain View, CA). DNA was isolated from saliva derived from Oragene DNA self-collection kits (DNA Genotek) customized for 23andMe. DNA extraction and genotyping were performed through 23andMe by the National Genetics Institute, a CLIA-certified clinical laboratory and subsidiary of Laboratory Corporation of America. One of two different Illumina arrays with custom content was used to provide genome-wide SNP data, the HumanOmniExpress or HumanOmniExpress-24^51–53^.

While genotype distribution did deviate from Hardy-Weinberg equilibrium across our entire sample (*χ* ^2^ = 8.13, *p* = 0.004), there was no deviating from equilibrium in our non-Hispanic Caucasian (*χ* ^2^ = 0.301, *p* = 0.583) or Asian (*χ* ^2^ = 0.311, *p* = 0.577) subsamples (the remaining subgroups did not yield sufficient number of individuals in one or more of the genotypes). There were 411 Val homozygotes, 210 heterozygotes, and 48 Met allele homozygotes. The heterozygotes and Met homozygotes were grouped together given the rarity of Met homozygotes. Genotype distributions were similar for the healthy subgroup (Supplementary Results). As expected, the allele frequency for the minor Met allele significantly differed as a function of race (see Table S2). Thus, to avoid conflation of race with genotype in our analyses, we adopted a two genotype group classification scheme: Val allele homozygotes and Met allele carriers. This classification scheme has been commonly employed in studies of rs6265. To address the potential confounding effects of race and ethnicity in our primary analyses, participant ancestry was modeled using the identity by state analysis in PLINK to extract the first four multidimensional scaling components for inclusion as covariates in all genotype analyses^54^.

### Statistical analysis

First, we used hierarchical regression analyses to test the relationship between the microstructural integrity of amygdala-vPFC pathways (FA values) and trait anxiety (STAI-T scores), while controlling for the effects of age, sex and head motion. Next, to test for a potential moderating effect of sex on the inverse correlation between the amygdala-vPFC pathway strength and trait anxiety, a moderator analysis was performed using the PROCESS macro^55^ implemented in SPSS 21 (IBM Corp., Armonk, NY, USA), controlling for age and head motion. To test for further moderating effects of rs6265 genotype, a second moderator analysis was carried out with sex and genotype (Val/Val vs. Met carriers) as moderators, controlling for age, ancestry, and head motion. Separate models were constructed and tested for left and right amygdala-vPFC pathways. All of the aforementioned analyses were rerun for participants with no current or past diagnoses for mental disorders (*n* = 541). For *post hoc* analyses, a false discovery rate correction was imposed on the significance threshold (*q* < 0.05) to correct for multiple statistical tests^56^.

### Data availability

The data that support the findings of this study are available on reasonable request from the corresponding author (see data sharing procedures through our website https://www.haririlab.com/projects/procedures.html). The data are not publicly available because they contain information that could compromise research participant privacy/consent.

## Electronic supplementary material

Supplementary Materials
