# Supplementary material for: Neurogenetic plasticity and sex influence the link between corticolimbic structural connectivity and trait anxiety
Source: Sci Rep. 2017 Sep 8;7:10959. doi: 10.1038/s41598-017-11497-2 (PMC5591318; doi:10.1038/s41598-017-11497-2)
Supplement: Supplementary file 1 — Supplementary Materials [file 41598_2017_11497_MOESM1_ESM.pdf]

# Neurogenetic plasticity and sex influence the link between corticolimbic structural connectivity and trait anxiety

M. Justin Kim, Reut Avinun, Annchen R. Knodt, Spenser R. Radtke & Ahmad R. Hariri

**- SUPPLEMENTARY INFORMATION -**

## SUPPLEMENTARY RESULTS

### Genotype Data for the Healthy Subgroup

Consistent with the whole group analysis, genotype distribution did deviate from Hardy-Weinberg equilibrium across our entire healthy subgroup ($\chi^2$ = 7.42, $p$ = 0.006), but not for non-Hispanic Caucasian ($\chi^2$ = 0.061, $p$ = 0.805) or Asian ($\chi^2$ = 0.217, $p$ = 0.642) subgroups (the remaining subgroups did not yield sufficient number of individuals in one or more of the genotypes). There were 326 Val allele homozygotes, 173 heterozygotes, and 42 Met allele homozygotes.

### Results for the Non-Hispanic Caucasian Subgroup

To test whether the findings from the whole group analysis were also observed in a subgroup with relatively homogeneous ancestry, the same analysis procedure was applied to non-Hispanic Caucasian participants only ($n$ = 276; healthy subgroup $n$ = 212). While the significance levels for some tests dropped, likely due to reduced power from smaller sample size, the overall findings were

consistent with and in the same direction as the whole group analysis. A full summary of the results is described below.

### *Genotype Data for the Non-Hispanic Caucasian Subgroup*

Genotype distribution did not deviate from Hardy-Weinberg equilibrium for the non-Hispanic Caucasian subgroup ($\chi^2$ = 0.3, $p$ = 0.583), as well as for the healthy subgroup ($\chi^2$ = 0.222, $p$ = 0.637). Across the entire non-Hispanic Caucasian subgroup, there were 180 Val allele homozygotes, 84 heterozygotes, and 12 Met allele homozygotes. As for the healthy subgroup, there were 139 Val allele homozygotes, 64 heterozygotes, and 9 Met allele homozygotes. Consistent with the whole group analysis, participants with the Val/Met and Met/Met allele were grouped together for further statistical analyses.

### *Demographic and Behavioral Characteristics*

A total of 276 participants identified themselves as being non-Hispanic Caucasian (142 women, ages 18-22 years, mean age = 19.7 years). Average STAI-T scores were 36.2 (± 8.7), and there were no significant difference between men and women. Among them, 212 did not have past or current diagnosis for mental disorders (113 females, ages 18-22 years, mean age = 19.7 years; see **Table S1** for full list of diagnoses). Average STAI-T scores for the healthy subsample were 35.3 (± 8), and once again there were no significant difference between men and women.

### *Amygdala-vPFC Structural Connectivity and Trait Anxiety*

Similar to the whole group analysis, when all non-Hispanic Caucasian participants (*n* = 276) were included, hierarchical regression analyses revealed that adding amygdala-vPFC pathway FA in the second step did not significantly improve the model from the first step that included age, sex, and head motion, in predicting self-reported trait anxiety (first step: $R^2 = 0.016$, $F(3, 272) = 1.464$, $p = 0.225$, second step, right pathway: $\Delta R^2 = 0.007$, $\Delta F(1, 271) = 1.821$, $\beta = -0.089$, $p = 0.178$; second step, left pathway: $\Delta R^2 = 0.012$, $\Delta F(1, 271) = 3.418$, $\beta = -0.122$, $p = 0.066$). When only healthy participants (*n* = 212) were included in the analysis, significant inverse correlations in the left pathway (first step: $R^2 = 0.012$, $F(3, 208) = 0.827$, $p = 0.48$, second step, left pathway: $\Delta R^2 = 0.022$, $\Delta F(1, 207) = 4.797$, $\beta = -0.167$, $p = 0.03$), but not in the right pathway (second step, right pathway: $\Delta R^2 = 0.011$, $\Delta F(1, 207) = 2.432$, $\beta = -0.118$, $p = 0.12$) were observed.

### *Sex Difference in the Present Brain-Anxiety Association*

A significant moderating effect of sex was observed for the right amygdala-vPFC pathway in predicting trait anxiety, after controlling for the effects of age and head motion ($\Delta R^2 = 0.019$, $F(1, 370) = 5.434$, $\beta = -0.148$, $p = 0.021$). Upon closer inspection, we found that the sex difference was driven by significant inverse association in women (first step: $R^2 = 0.011$, $F(2, 139) = 0.772$, $p = 0.464$, second step, right pathway: $\Delta R^2 = 0.055$, $\Delta F(1, 138) = 8.105$, $\beta = -0.244$, $p = 0.005$, $q < 0.05$). The left amygdala-vPFC pathway, while the moderator analysis was not significant, still showed significant inverse association in women

(second step, left pathway: $\Delta R^2 = 0.047$, $\Delta F(1, 138) = 6.87$, $\beta = -0.221$, $p = 0.01$), but not in men.

Results were consistent when the analysis only included healthy participants, as a significant moderating effect of sex was observed for the right amygdala-vPFC pathway in predicting trait anxiety ($\Delta R^2 = 0.019$, $F(1, 206) = 4.065$, $\beta = -0.132$, $p = 0.045$). Similarly, this sex difference was driven by significant inverse association in women (first step: $R^2 = 0.022$, $F(2, 110) = 1.209$, $p = 0.302$, second step, right pathway: $\Delta R^2 = 0.052$, $\Delta F(1, 109) = 6.615$, $\beta = -0.256$, $p = 0.011$, $q < 0.05$), but not in men. The left amygdala-vPFC pathway, while the moderator analysis was not significant, still showed significant inverse association in women (second step, right pathway: $\Delta R^2 = 0.049$, $\Delta F(1, 109) = 5.786$, $\beta = -0.231$, $p = 0.018$) but not in men.

### *Moderating Effect of BDNF rs6265 Genotype and Sex on the Present Brain-Anxiety Association*

There were no overall significant effects of BDNF genotype on the present brain-anxiety association. However, a significant moderating effect of *BDNF* rs6265 genotype × sex interaction was observed for the right amygdala-vPFC pathway in predicting trait anxiety, after controlling for the effects of age, ancestry (the first two MDS components instead of four was used, since the analysis was restricted to the non-Hispanic Caucasian subsample), and head motion ($\Delta R^2 = 0.033$, $F(3, 264) = 3.093$, $p = 0.028$). *Post hoc* correlation tests showed that this effect was driven by an inverse correlation in women carrying the Met allele (first step: $R^2$ =

0.045, $F(4, 44) = 0.52$, $p = 0.721$, second step, right pathway: $\Delta R^2 = 0.099$, $\Delta F(1, 43) = 4.991$, $\beta = -0.337$, $p = 0.031$, but $q > 0.05$) and a positive correlation in men carrying the Met allele (first step: $R^2 = 0.023$, $F(4, 42) = 0.247$, $p = 0.302$, second step, right pathway: $\Delta R^2 = 0.14$, $\Delta F(1, 41) = 6.881$, $\beta = 0.414$, $p = 0.012$, $q < 0.05$; ; **Figure S1**), but not for all other groups. This moderating effect was not observed in the left amygdala-vPFC pathway.

This interactive effect of *BDNF* rs6265 genotype and sex on the right amygdala-vPFC pathway was observed when the data were reanalyzed just including healthy participants ($\Delta R^2 = 0.041$, $F(3, 200) = 2.984$, $p = 0.032$). *Post hoc* tests showed that a trend for an inverse correlation was found in women with the Met allele (first step: $R^2 = 0.163$, $F(4, 33) = 1.601$, $p = 0.197$, second step, right pathway: $\Delta R^2 = 0.084$, $\Delta F(1, 32) = 6.881$, $\beta = -0.311$, $p = 0.067$), and a positive correlation in men carrying the Met allele (first step: $R^2 = 0.022$, $F(4, 30) = 0.167$, $p = 0.953$, second step, right pathway: $\Delta R^2 = 0.213$, $\Delta F(1, 29) = 8.075$, $\beta = 0.527$, $p = 0.008$, $q < 0.05$; **Figure S2**). A summary of the pairwise correlation tests for all sub groups is provided in **Table S3**.

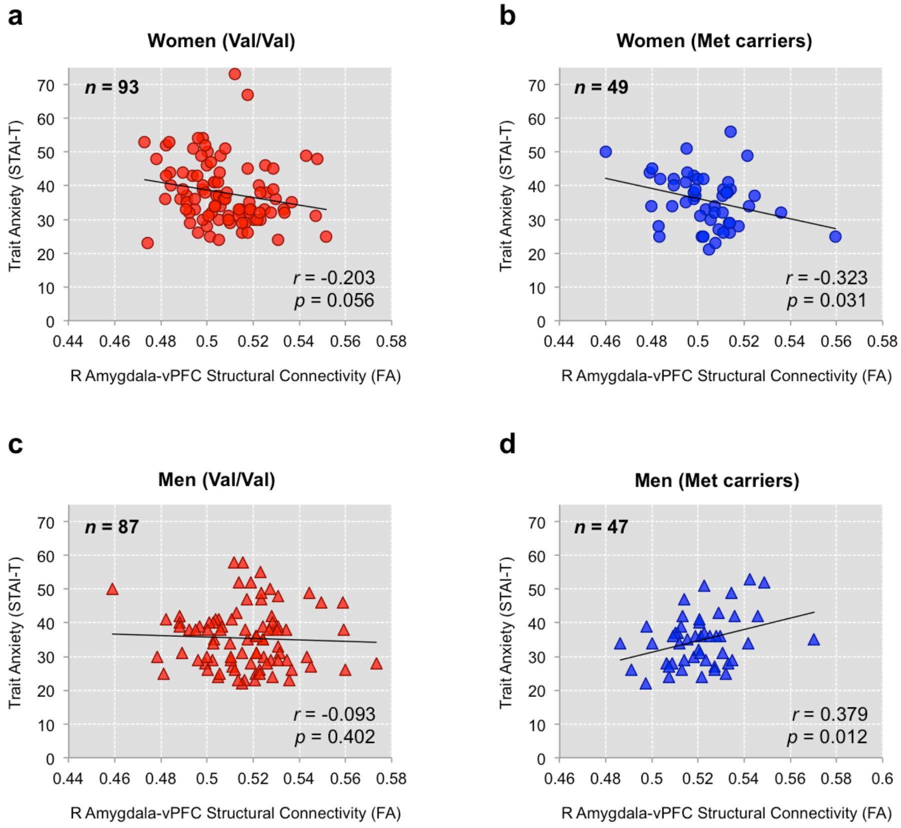


**Figure S1.** Moderating effect of rs6265 genotype on sex-dependent brain-anxiety association within the non-Hispanic Caucasian subsample ($n$ = 276). (a-d) Consistent with the analysis in all participants, women Met allele carriers (blue circle) displayed an inverse correlation between the structural integrity of the right amygdala-vPFC pathway and trait anxiety, whereas men Met allele carriers (blue triangle) displayed a positive correlation. All correlation coefficients were calculated controlling for the effects of age, ancestry, and head motion.

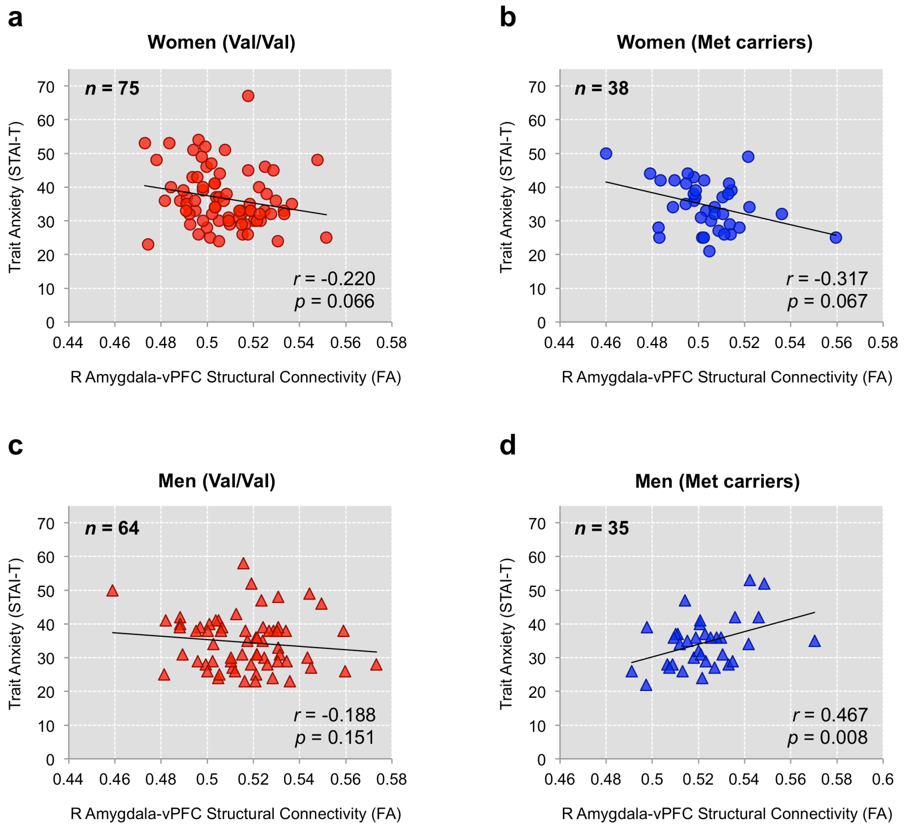


**Figure S2.** Moderating effect of rs6265 genotype on sex-dependent brain-anxiety association when the analyses were limited to healthy non-Hispanic Caucasian subsample (*n* = 212). (a-d) Consistent with the analysis in all participants, women Met allele carriers (blue circle) displayed an inverse correlation between the structural integrity of the right amygdala-vPFC pathway and trait anxiety at a trend level, whereas men Met allele carriers (blue triangle) displayed a positive correlation. All correlation coefficients were calculated controlling for the effects of age, ancestry, and head motion.

**Table S1.** Diagnoses for past or current mental disorders in the total study sample (*n* = 128) and the non-Hispanic Caucasian subgroup (*n* = 64).

| Diagnosis | Total | Non-Hispanic Caucasian |
|---|---|---|
| Major depressive disorder | 26 | 12 |
| Bipolar disorder I or II | 5 | 3 |
| Bipolar disorder – Not otherwise specified | 11 | 8 |
| Hypomanic episode | 12 | 4 |
| Panic disorder | 8 | 5 |
| Agoraphobia | 10 | 8 |
| Social anxiety disorder | 7 | 2 |
| Generalized anxiety disorder | 13 | 6 |
| Obsessive-compulsive disorder | 6 | 3 |
| Posttraumatic stress disorder | 1 | 0 |
| Alcohol abuse/dependence | 75 | 42 |
| Marijuana abuse/dependence | 23 | 10 |
| Eating disorder | 5 | 2 |
| Antisocial personality disorder | 1 | 0 |
| Borderline personality disorder | 1 | 0 |

The sum of the individual diagnoses is higher than the number of participants with mental disorders because a subsample of them has multiple comorbid diagnoses

**Table S2.** *BDNF* rs6265 genotypes as a function of race/ethnicity

| **Race** | **Val/Val** | | **Val/Met** | | **Met/Met** | |
|---|---|---|---|---|---|---|
| | Men | Women | Men | Women | Men | Women |
| Non-Hispanic Caucasian | 87 | 93 | 40 | 44 | 7 | 5 |
| Black/ African American | 17 | 56 | 1 | 2 | 0 | 0 |
| Asian | 26 | 40 | 39 | 44 | 14 | 17 |
| American Indian/ Alaska Native | 2 | 0 | 0 | 0 | 0 | 0 |
| Multiracial | 12 | 19 | 9 | 8 | 0 | 4 |
| Other | 4 | 11 | 3 | 3 | 0 | 0 |
| Hispanic Caucasian | 26 | 18 | 7 | 10 | 0 | 1 |
| Total | 174 | 237 | 99 | 111 | 21 | 27 |

**Table S3.** Summary of correlation coefficients for each sex × BDNF genotype subgroups within the non-Hispanic Caucasian subsample. Coefficients were calculated controlling for the effects of age, ancestry, and head motion.

| **Non-Hispanic Caucasian Subsamples** | | **R Amygdala-vPFC – STAI-T Correlation** | **L Amygdala-vPFC – STAI-T Correlation** |
|---|---|---|---|
| **Total ($n$ = 276)** | | | |
| Women ($n$ = 142) | Val/Val ($n$ = 93) | $r$ = -0.203 $p$ = 0.056 | $r$ = -0.223 $p$ = 0.036 |
| | Met Carriers ($n$ = 49) | $r$ = -0.323 $p$ = 0.031 | $r$ = -0.235 $p$ = 0.120 |
| Men ($n$ = 134) | Val/Val ($n$ = 87) | $r$ = -0.093 $p$ = 0.402 | $r$ = -0.146 $p$ = 0.189 |
| | Met Carriers ($n$ = 47) | $r$ = 0.379 $p$ = 0.012 | $r$ = 0.168 $p$ = 0.282 |
| **Healthy ($n$ = 212)** | | | |
| Women ($n$ = 113) | Val/Val ($n$ = 75) | $r$ = -0.220 $p$ = 0.066 | $r$ = -0.238 $p$ = 0.046 |
| | Met Carriers ($n$ = 38) | $r$ = -0.317 $p$ = 0.067 | $r$ = -0.257 $p$ = 0.142 |
| Men ($n$ = 99) | Val/Val ($n$ = 64) | $r$ = -0.188 $p$ = 0.151 | $r$ = -0.268 $p$ = 0.038 |
| | Met Carriers ($n$ = 35) | $r$ = 0.467 $p$ = 0.008 | $r$ = 0.232 $p$ = 0.209 |

**Table S4**. Summary of correlation coefficients for each sex × BDNF genotype subgroups for all participants. Coefficients were calculated controlling for the effects of age, ancestry, and head motion.

| **Study Group** | | **R Amygdala-vPFC – STAI-T Correlation** | **L Amygdala-vPFC – STAI-T Correlation** |
|---|---|---|---|
| **Total ($n$ = 669)** | | | |
| Women ($n$ = 375) | Val/Val ($n$ = 237) | $r$ = -0.078 $p$ = 0.237 | $r$ = -0.161 $p$ = 0.014 |
| | Met Carriers ($n$ = 138) | $r$ = -0.229 $p$ = 0.008 | $r$ = -0.130 $p$ = 0.137 |
| Men ($n$ = 294) | Val/Val ($n$ = 174) | $r$ = 0.092 $p$ = 0.235 | $r$ = 0.049 $p$ = 0.527 |
| | Met Carriers ($n$ = 120) | $r$ = 0.212 $p$ = 0.024 | $r$ = 0.147 $p$ = 0.120 |
| **Healthy ($n$ = 541)** | | | |
| Women ($n$ = 318) | Val/Val ($n$ = 201) | $r$ = -0.112 $p$ = 0.120 | $r$ = -0.203 $p$ = 0.004 |
| | Met Carriers ($n$ = 117) | $r$ = -0.305 $p$ = 0.001 | $r$ = -0.222 $p$ = 0.019 |
| Men ($n$ = 223) | Val/Val ($n$ = 125) | $r$ = -0.002 $p$ = 0.979 | $r$ = -0.015 $p$ = 0.873 |
| | Met Carriers ($n$ = 98) | $r$ = 0.202 $p$ = 0.054 | $r$ = 0.203 $p$ = 0.052 |
